# Does the Time of Drug Administration Alter the Metabolic Risk of Aripiprazole?

**DOI:** 10.3389/fpsyt.2018.00494

**Published:** 2018-10-11

**Authors:** Danielle A. Chipchura, Zachary Freyberg, Corey Edwards, Susan G. Leckband, Michael J. McCarthy

**Affiliations:** ^1^VA San Diego Healthcare System, Mental Health Service, San Diego, CA, United States; ^2^Department of Psychiatry and Cell Biology, University of Pittsburgh, Pittsburgh, PA, United States; ^3^Department of Psychiatry and Center for Circadian Biology, University of California, San Diego, San Diego, CA, United States

**Keywords:** antipsychotic, diabetes mellitus, metabolism, circadian rhythm, weight gain, cholesterol, dopamine

## Abstract

Antipsychotic drugs cause metabolic abnormalities through a mechanism that involves antagonism of D_2_ dopamine receptors (D_2_R). Under healthy conditions, insulin release follows a circadian rhythm and is low at night, and in pancreatic beta-cells, D_2_Rs negatively regulate insulin release. Since they are sedating, many antipsychotics are dosed at night. However, the resulting reduction in overnight D_2_R activity may disrupt 24 h rhythms in insulin release, potentially exacerbating metabolic dysfunction. We examined retrospective clinical data from patients treated over approximately 1 year with the antipsychotic drug aripiprazole (ARPZ), a D2R partial agonist. To identify effects of timing on metabolic risk, we found cases treated with ARPZ either in the morning (*n* = 90) or at bedtime (*n* = 53), and compared hemoglobin A1c, and six secondary metabolic parameters across the two groups. After controlling for demographic and clinical factors, patients treated with ARPZ at night had a significant decrease in HDL cholesterol, while in patients who took ARPZ in the morning had no change. There was a non-significant trend toward higher serum triglycerides in the patients treated with ARPZ at night vs. morning. There were no group differences in hemoglobin A1c, BMI, total cholesterol, LDL cholesterol, or blood pressure. Patients taking APPZ at night developed a worse lipid profile, with lower HDL cholesterol and a trend toward higher triglycerides. These changes may pose additional metabolic risk factors compared to those who take ARPZ in the morning. Interventions based on drug timing may reduce some of the adverse metabolic consequences of antipsychotic drugs.

## Introduction

Serious mental illnesses (SMI) including Bipolar Disorder, Major Depression, and Schizophrenia are commonly treated with antipsychotic medications. While these drugs differ in their pharmacological mechanisms and clinical profiles, all are associated with weight gain, metabolic changes in glucose and lipid homeostasis, hypertension and increased risk for non-insulin dependent (type 2) diabetes mellitus (T2D)(1). SMI is associated with early mortality, in large part due to metabolic disease (2–4). The mechanisms underlying this elevated risk are multifactorial. Considerable evidence reveals that the presence of SMI may be an independent risk factor for the development of insulin resistance and metabolic disease (5, 6). When examined in healthy controls in laboratory settings, antipsychotic drugs have direct effects on glucose metabolism in Burghardt et al. (7). Even drugs considered “metabolically neutral” like aripiprazole (ARPZ) may promote glucose intolerance (8). Therefore, the metabolic consequences of antipsychotic drugs may further exacerbate metabolic risk in the SMI population that is already vulnerable by its predisposition to metabolic diseases. Accordingly, induced weight gain is a major limitation of antipsychotic drug treatment, and a common reason for premature discontinuation (9). There is an important need to reduce the metabolic burden on SMI patients treated with antipsychotics.

Circadian mechanisms may contribute to antipsychotic-induced metabolic disturbances. Circadian clocks in the brain, liver, adipocytes, and endocrine organs serve important roles in regulating feeding behaviors, activity, glucose homeostasis, and lipid metabolism (10–14). Insulin release is regulated by the circadian clock, with levels that peak during the day, anticipating the food intake and higher levels of blood sugar that typically occur during wakefulness. At night, insulin levels are low, facilitating the breakdown of lipid and glycogen stores to maintain glucose homeostasis during the fasting period associated with sleep (15). In mutant mice with pancreatic beta cell-specific knockout of the circadian clock, animals gain weight and develop insulin resistance, phenotypes that resemble non-insulin dependent T2D in humans (11). Similarly, humans with T2D show disrupted circadian rhythms in insulin release (16).

Among its many functions, the circadian clock also regulates dopamine, including its biosynthesis (17), the release of striatal dopamine in the CNS (18), and the expression of dopamine receptors (19). Interestingly, dopamine is also important in the pancreas to negatively regulate the release of insulin by beta islet cells (20). By blocking the D_2_/D_3_ dopamine receptors, antipsychotic drugs have potent effects on glucose stimulated insulin release, leading to disinhibition of insulin signaling, greater overall release in response to a glucose challenge, and decreasing insulin-stimulated glucose uptake (21, 22).

Due to their observed effects on beta cells and increasing insulin release, we hypothesized that an antipsychotic drug taken when insulin levels are high in the morning would show a more favorable metabolic profile (especially glucose levels) compared to the same drug taken in the evening when insulin levels are low (14). To test this hypothesis, we used a naturalistic, retrospectively ascertained cohort to compare patients who took aripiprazole (ARPZ), an antipsychotic drug that selectively targets the D_2_ dopamine receptor (D_2_R) in the morning against those who took ARPZ at night. Hemoglobin A1c (HbA1c) was used as the primary endpoint to estimate changes in glucose sensitivity. Lipid profile, body mass index (BMI) and blood pressure were examined as secondary measures of metabolic health.

## Materials and methods

### Study design

Using pharmacy records at the VA San Diego Healthcare System (VASDHS), we conducted a retrospective chart review to compare long term metabolic outcomes in veterans taking ARPZ in the morning vs. those taking ARPZ at night. Data from 14 years (January 1, 2002 to December 31, 2016) were included in the analysis. The research was reviewed and approved by the VASDHS IRB to ensure compliance with all pertinent regulations regarding human subjects research.

### Drug selection and dosing

ARPZ was previously considered metabolically neutral, but recently has been associated with weight gain and metabolic disturbances (23–25). Therefore, ARPZ was expected to be informative, with a wide range of metabolic outcomes, and perhaps sensitive to differences in dosing schedule. Moreover, while many antipsychotic drugs target a variety of receptors, the mechanism of ARPZ is relatively selective as a partial agonist at D_2_R. Finally, large numbers of patients take ARPZ at different times across the day, while other drugs we considered (e.g., olanzapine, risperidone, perphenazine, haloperidol) were strongly biased toward night or day, and/or prescribed in insufficient numbers to yield a suitable cohort after applying exclusion criteria. For each subject, pharmacy prescription orders were analyzed. Subjects were considered to have taken the drug in the morning if the instructions indicated morning, daily, qDAY or qAM. Subjects were considered to have taken the drug at night when the instructions indicated qHS or bedtime. Only oral ARPZ was considered. Daily doses ranging from 2 to 30 mg were included. In secondary analyses of dose, subjects taking 2 and 5 mg were consolidated into a single group, as were subjects taking 15 and 20 mg to account for the small number of subjects taking 2 and 15 mg doses. Those taking injectable ARPZ or any other antipsychotic drugs (oral or injectable) were excluded. Compliance with ARPZ was estimated by the frequency of on time refills requested by the patient. Medication compliance was defined as a proportion of days covered > 0.8.

### Subjects

All subjects were veterans receiving standard clinical care for SMI at the VASDHS. The study included adult patients (age 18–75 years) taking ARPZ for a range of SMI indications including psychotic disorders [schizophrenia (SCHZ), schizoaffective disorder (SAD), or unspecified psychosis], bipolar disorders (BD), including bipolar I, bipolar II, unspecified bipolar disorder, and depressive disorders [major depressive disorder (MDD), MDD with psychotic features, post-traumatic depression and any unspecified depression]. Comorbid PTSD was pervasive in our cohort, but not considered a primary indication for ARPZ. Subjects with a diagnosis of Alzheimer's disease, Parkinson's disease, dementia or other neurocognitive disorder were excluded from consideration. Subjects with existing metabolic disorders including T2D, essential hypertension, and hypercholesterolemia were included.

### Selection of study cohort

Study subjects were selected based on their use of long-term ARPZ with confirmed compliance. We sought subjects taking the medication for approximately 1 year. The index date was defined as the first day that ARPZ was released to the patient and the target end date was defined as the index date plus 365 days. Subjects were included in the study if two HbA1c reading were present, one within 3 months of the start of ARPZ, and one 9–15 months later (i.e., target end date ± 3 months). Further inclusion criteria were compliance with clinical and laboratory screening for metabolic changes, both at baseline and at the end of the 1-year study period, covering HbA1c, serum glucose, fasting lipid panel, blood pressure, weight and BMI.

### Data collection

As outcome measures, we selected one primary variable, change in HbA1c, and six related secondary variables: weight gain, total cholesterol, HDL cholesterol, LDL cholesterol, triglycerides, blood pressure. Height was recorded to allow calculation of BMI, but was not considered as an outcome variable, as it was not expected to change as a result of drug treatment. Demographic variables (age, sex, and race) and clinical data (dose of ARPZ, hypertension, use of metformin, use of a statin drug) were considered as potential modifiers. Also considered as modifiers were medications commonly used in SMI that significantly affect weight (valproic acid, mirtazapine, and topiramate). Smoking, alcohol and substance use history were not reliably recorded in the pharmacy database and were not included. Only data sets including complete baseline and follow-up HbA1c, lipid panel, and blood pressure measures were used. Missing data for BMI was allowed (applied to 34 subjects).

### Statistical analyses

A one-way analysis of covariance (ANCOVA) comparing morning versus nighttime APRZ groups for change in individual metabolic parameters, using age, dose, sex, and race as covariates. For univariate analyses of categorical measures, we used a chi squares test. Additional *post-hoc* tests were performed using ANOVA or *t*-tests. Statistical significance for each variable was considered as *p* < 0.05. Analyses were conducted using SPSS version 20 (IBM, Armonk NY).

## Results

After applying inclusion and exclusion criteria, our initial screen identified 1,009 subjects treated for 1 year with ARPZ that had at least one HbA1c reading during the treatment period. However, only 143 subjects had complete data that were suitable for analysis (i.e., data for each metabolic measure at the start and end of the study period). Of these, a majority (*n* = 90) were treated with oral ARPZ in the morning and the remainder (*n* = 53) were treated with ARPZ at night. The mean time of follow-up was similar for both groups (slightly longer than 1 year, Table 1). The majority of subjects in both groups were male, but there was no significant difference between groups in gender distribution. Age, racial background, treatment for diabetes, treatment for hypercholesterolemia, and the use of weight altering medications was similar between groups (Table 1). The average dose (mean ±SEM) of ARPZ used in each group was 15.1 ± 0.9 mg for the morning group, and 12.1 ± 1.3 mg for the night group, reflecting the fact that the morning group was more likely to get ARPZ doses of 10-20 mg, while the nighttime group was more like to get doses of 2–5 mg. These differences were statistically significant (Table 1).

**Table 1:** Demographic characteristics of study sample.

**Characteristic**	**Morning *N* = 90**	**Bedtime *N* = 53**	***P*-value**
**AGE**			
Age range (Years)	30–73	24–67	
Age (mean ± SEM)	53.46 ± 1.1	53.26 ± 1.2	NS
Male sex, n (%)	79 (87.7)	49 (92.4)	NS
**FOLLOW-UP PERIOD**			
Range (days)	249–554	295–552	
Observation days (Mean ± SEM)	385 ± 6.1	400 ± 9.4	NS
**DIAGNOSIS**			
Psychotic disorder (%)	11 (21)	22 (24)	NS
Depressive disoder (%)	28 (53)	42 (47)	
Bipolar disorder (%)	14 (26)	26 (29)	
**RACE**			
Caucasian, n (%)	56 (62)	31 (58)	NS
African American, n (%)	17 (19)	9 (17)	
Asian American, n (%)	6 (7)	6 (11)	
Other/Unknown, n (%)	11 (12)	6 (11)	
**DOSE ARIPIPRAZOLE**, ***N*** **(%)**			
Average Dose (Mean ± SEM)	15.2 ± 0.9	12.1 ± 1.3	0.05*
2–5 mg, *n* (%)	14 (16)	27 (51)	
10 mg, *n* (%)	32 (36)	9 (17)	
15–20 mg, *n* (%)	24 (27)	8 (15)	
30 mg, *n* (%)	16 (18)	9 (17)	
**OTHER MEDICAL FACTORS**			
Diagnosis hypertension, *n* (%)	51 (57)	36 (68)	NS
Mirtazapine use, n (%)	7 (8)	4 (8)	NS
Metformin use, n (%)	2 (2)	0 (0)	NS
Statin use, n (%)	7 (8)	1 (2)	NS

**Figure 1:**
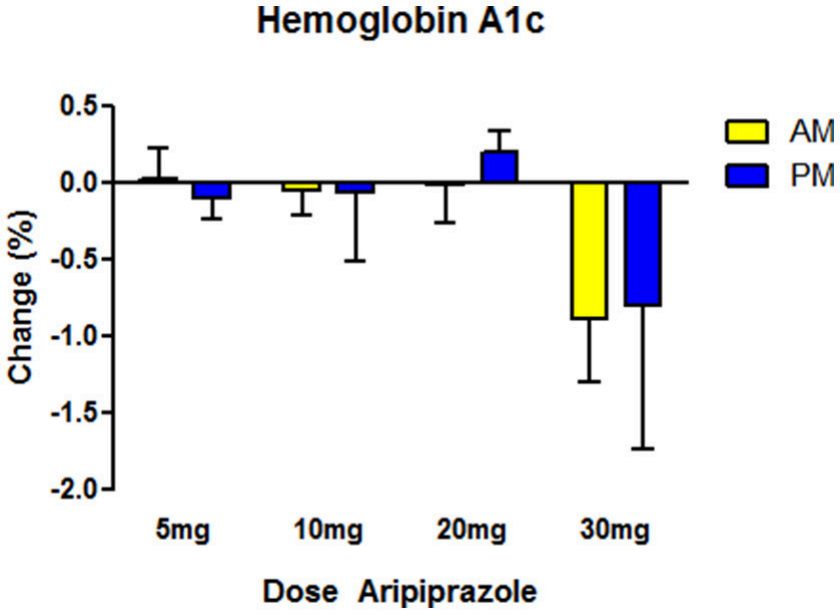
Effects on glucose homeostasis as measured by hemoglobin A1c (HbA1c) glycosylation after treatment for ~1 year with aripiprazole. The highest dose (30 mg) was associated with reductions in HbA1c in both the morning (AM) and night time (PM) group (Two-Way ANOVA, dose *p* < 0.05, Time NS, interaction NS). Time of administration made no difference in HbA1c. For morning group, *N* = 90 with *N* = 14–36 in each dose category. For night time, *N* = 53, with *N* = 8–27 in each dose category. Data indicate mean values of post minus pre HbA1c readings. Error bars reflect standard error of the mean (SEM).

Baseline HbA1c levels were 6.69 ± 0.15% for the morning group and 6.25 ± 0.22% for the night group (mean ± SEM), values that exceed the upper healthy limit (5.7%). In the morning group 36% (33/90) had HbA1c ≥6.5% (cutoff value for probable D2M). In the night group, the number was similar 33% (18/53), indicating both groups showed evidence of poor glycemic control, and were at high risk for D2M and other metabolic disorders (Table 2). Over the course of treatment, there was a statistically insignificant reduction in mean HbA1c levels in both groups, and significant effect of ARPZ dose, with higher doses significantly associated with greater HbA1c reduction (Figure 1). However, regarding time of dosing and change in HbA1c, our primary outcome variable, there were no significant differences between daytime and nighttime schedules either nominally or after adjusting for age, dose, sex, and race.

**Table 2:** Metabolic parameters before/after treatment with oral aripiprazole.

**Outcome**	**Morning (AM)**	**Bedtime (PM)**	***P*-value unadjusted**	***P*-value adjusted**
**FOLLOW-UP PERIOD**				
Range (days)	249–554	295–552		
Observation days (Mean ± SEM)	385 ± 6.1	400 ± 9.4	NS	NS
**BLOOD GLUCOSE**				
Baseline HbA1c (% glycosylated)	6.7 ± 0.2	6.4 ± 0.2	NS	NS
Change HbA1c (% glycosylated)	−0.2 ± 0.1	−0.2 ± 0.2	NS	NS
HbA1c ≥ 5.7% at baseline, number (%)	33 (37%)	18 (34%)	NS	NS
**BODY WEIGHT**				
Baseline BMI (kg/m^2^)	33.4 ± 6.9	32.9 ± 6.6	NS	NS
Change BMI (kg/m^2^)	0.85 ± 2.3	0.6 ± 1.9	NS	NS
**SERUM CHOLESTEROL (MG/DL)**				
Baseline total cholesterol	184.4 ± 5.8	183.7 ± 6.2	NS	NS
Change total cholesterol	−5.3 ± 5.1	−2.5 ± 6.2	NS	NS
Baseline LDL cholesterol	102.4 ± 4.2	103.3 ± 5.4	NS	NS
Change LDL cholesterol	−5.9 ± 43.4	−2.8 ± 36.6	NS	NS
Baseline HDL cholesterol	43.2 ± 1.3	46.9 ± 2	NS	NS
Change HDL cholesterol	0.2 ± 0.9	−3.8 ± 1.3	0.009	0.04^*^
Baseline triglycerides	221 ± 1	166 ± 7	NS	NS
Change triglycerides	22124.6 ± 13.2	31.6 ± 12.0	0.06	0.11
**BLOOD PRESSURE (mmHg)**				
Baseline systolic	130 ± 18.6	129 ± 16.6	NS	NS
Change systolic	−2.57 ± 20.8	−1.4 ± 20	NS	NS
Baseline diastolic	78 ± 14.3	80 ± 11.4	NS	NS
Change diastolic	−2.4 ± 16.8	−2.6 ± 15.9	NS	NS

* *statistically significant after adjusting for age, sex, dose, race*.

**Figure 2:**
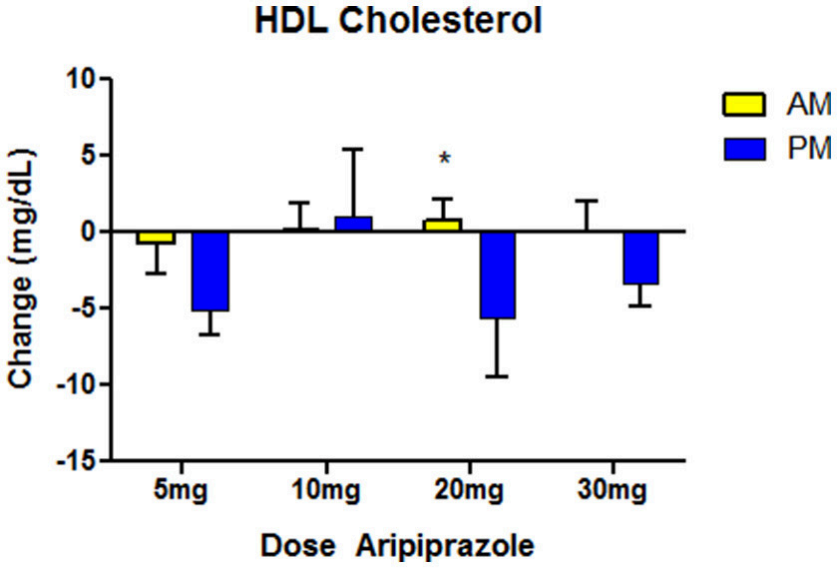
Effects on HDL cholesterol after ~1-year treatment with aripiprazole. Time of administration made a significant difference in HDL levels at ~1-year follow-up, with reductions seen only in the night time (PM), and not in the morning (AM) group. The difference was nominally present at most doses, but only the 20 mg dose showed a significant difference in a *post-hoc T*-test (* indicates *p* < 0.05 indicates). For morning group N = 90, with N = 14-36 in each dose category. For night time group, *N* = 53 PM, with *N* = 8–27 in each dose category. Data indicate mean values of post minus pre-HDL readings. Error bars reflect standard error of the mean (SEM).

Among the secondary measures, serum HDL showed a significant reduction in the night dosing group compared to the morning group (Table 2). The effects remained significant after adjusting for age, dose, sex, and race. In *post-hoc* tests we found that the effects of timing on HDL cholesterol were nominally present across the entire dose range (5–30 mg), but reached statistical significance only at the 20 mg dose (Figure 2). Serum triglycerides showed a non-significant trend toward increase in the nighttime group compared to the daytime group (Table 2). There were no differences between nighttime and daytime ARPZ dosing in the other outcome measures: total cholesterol, BMI, LDL, diastolic, and systolic blood pressure (Table 2).

## Discussion

Food intake, sleep and metabolism are tightly coordinated and interrelated physiological processes connected by two common features: the circadian clock and dopamine signaling (14). The circadian clock strongly influences the 24 h. sleep-wake cycle, and through this effect on activity on requisite caloric expenditure, dictates the energy needs of the body. Accordingly, glycogenolysis, lipolysis, adipogenesis, and numerous other bioenergetic processes also follow circadian rhythms (15). Disruption of rhythms in experimental animals causes hyperglycemia, hyperlipidemia, and other metabolic consequences (10–13), and in humans, T2D is associated with altered rhythms in insulin release (16). Dopamine is one of the signaling systems that link brain activity to peripheral metabolism (26). The mesolimbic/mesocortical dopamine systems are involved in goal directed activity and arousal. These forebrain dopamine systems stimulate wakefulness and feeding behaviors in response to stimuli such as orexins, serotonin, and neuropeptides. In the periphery, dopamine negatively regulates insulin release from pancreatic beta cells and leptin from adipocytes (27, 28). Dopaminergic agonists such as bromocriptine suppress insulin and leptin release by reducing peak amplitude of their rhythmic release, and have been associated with improved metabolic function in obese subjects with metabolic syndrome, and subjects suffering from T2D (29, 30).

For the reasons above, we hypothesized that antagonism of the D_2_R by ARPZ would have differential effects on metabolism depending on the circadian cycle, and the time of drug administration. We expected this effect to emerge independently of master clock in the suprachiasmatic nucleus which is largely depleted in D_2_R (14). Instead, we expected the effect to emerge from the peripheral action of ARPZ on D_2_R and D_3_R in islet beta cells, thereby increasing overnight insulin release, with subsequent flattening of the insulin rhythm (i.e., amplitude reduction). Given the retrospective nature of the study, we were not able to assess insulin directly, and relied upon HbA1c as a proxy. Perhaps because of the nature of HbA1c, which averages blood glucose levels over weeks, we failed to detect a time of day dependent difference in HbA1c, our primary endpoint. However, we did find differences in HDL cholesterol associated with the time of ARPZ administration. Low HDL cholesterol is a risk factor for cardiovascular disease (31). The decrease in serum HDL observed exclusively in the ARPZ night treated group indicates this risk factor was exacerbated over the course of treatment selectively in the night group while remaining constant in the morning group. This finding could indicate that the metabolic consequences of ARPZ, and perhaps other antipsychotic drugs could be mitigated or worsened by the administration schedule. As metabolic side effects are a major limitation to the use of antipsychotics in SMI, the ability to reduce this risk would mark an important advance.

Based on previous literature, we hypothesize that nightly dosing with ARPZ could interfere with typical rhythms of insulin or leptin release that typically reach nadir overnight in human subjects. Elevated insulin/leptin at night may lead to reduced lipolysis or similar catabolic adaptations to provide the body glucose during overnight fasting. Insulin stimulates triglyceride accumulation, a key mechanism involved in reducing HDL in T2D (32). While not statistically significant, we did observe a nominal difference toward elevated triglycerides in patients treated at night with ARPZ. Cholesterol levels, including HDL cholesterol has been show to oscillate over the day in a circadian manner, with higher amplitude oscillations in total cholesterol associated with greater longevity (33). T2D subjects with a strong predisposition toward night time activity (evening chronotypes) show lower levels of HDL cholesterol, and elevated triglycerides (34), a factor that may contribute to the excess mortality observed in evening chronotypes in the general population (35). Therefore, we propose that one explanation for the observed elevations in triglyceride, and reductions in HDL could be due to ARPZ disrupting rhythms in insulin.

We conducted our study in a population of veterans with SMI who were chronically treated with ARPZ for approximately 1 year. The long-term follow-up data for our subjects including laboratory monitoring and verified compliance are important strengths of our work. However, this study population was distinctive in several key respects and may not generalize to other groups. ARPZ is not available as a first line medication at the VASDHS and requires prior authorization from the pharmacy. Therefore, the vast majority of subjects in our study were older, male, and already receiving one or more antipsychotic medications prior to receiving ARPZ. Indeed, they may have been preferentially selected for treatment with ARPZ because they experienced metabolic side effects as a result of prior treatments with another antipsychotic drug. The high incidence in our study of hyperglycemia at baseline, and the trend toward metabolic improvement in some domains supports this interpretation. Notably, the ARPZ morning group received higher average doses of medication compared to the nighttime group, despite the fact that high dose ARPZ is more likely to be sedating (36). This could speak to possible group differences in activity or sleep that were not assessed using our approach. Future prospective studies conducted in drug naïve subjects would be informative.

ARPZ has a long half-life of approximately 75 h. and active metabolites (e.g., dehydro-ARPZ) with even longer half-lives (>94 h). Therefore, steady state drug levels are unlikely to explain our findings. However, the major metabolic pathways affecting metabolism of ARPZ in the liver, CYP2D6 and CYP3A4 have been shown to oscillate under the regulation of the circadian clock and it remains possible that one or more ARPZ metabolites may fluctuate over the circadian time course (37, 38). Perhaps more importantly, peak ARPZ levels and pharmacological effects occur rapidly after oral administration, and in the mouse, mesolimbic dopamine release and the expression of both D2 and D3 dopamine receptors are rhythmic (18, 19). Therefore, the primary drug targets of ARPZ may oscillate independently of drug levels, and despite the long half-life of ARPZ, there may be important pharmacodynamic consequences of drug administration, particularly as it relates to time-sensitive processes like insulin rhythms.

Our data must be interpreted with a number of considerations and caveats in mind. ARPZ has an unusual mechanism of action, working primarily as a partial agonist at D_2_R and D_3_R, but also has affinity for serotonin receptors including 5-HT1A, 5-HT2A, 5-HT2B, and 5-HT7, effects that can modulate dopamine indirectly (39). As the drug is neither fully inhibitory nor excitatory at dopamine receptors, conclusions about the metabolic effects of dopamine antagonism across the circadian cycle must be made cautiously. We interpret our data in the context of a substantial body of work that suggests D_2_R blockade is the common mechanism across all antipsychotic drugs, all of which cause weight gain to some extent, and accordingly that blockade of dopamine receptors is likely an important mechanism underlying metabolic dysfunction. However, future studies addressing this hypothesis should use drugs with a simpler mechanism to more clearly delineate this relationship.

Our study design had a number of important limitations. First, the study was retrospective without random assignment. The stringency of our inclusion criteria reduced the sample size, making the sample insufficiently powered to address some key issues such as gender differences and the role of smoking, alcohol and other key clinical variables. Next, we relied on pharmacy records to determine dosing schedules, and were unable to verify that our subjects took the medication at the stated time. Finally, there was significant heterogeneity in our sample in psychiatric diagnoses, duration of illness, prior medication history, and medical comorbidity. Nonetheless, we contend that this work marks an important preliminary step in identifying circadian mechanisms underlying antipsychotic induced metabolic dysfunction. Future studies with uniform inclusion/exclusion criteria, random assignment, prospective assessment and detailed laboratory analyses of diurnal insulin levels, glucose tolerance, and lipid profiles are warranted. If empirically supported, manipulations to the dosing schedule of antipsychotic drugs could point the way to relatively simple, and cost-effective methods to reduce the medical comorbidity associated with antipsychotic drugs in the treatment of SMI.

### Previous presentation

Oral presentation at the 2017 VA VISN 22 Conference for Pharmacy Residents and Preceptors in San Diego, CA. Poster at the 2017 Annual Meeting of the Society for Neuropsychopharmaocology, Palm Desert CA.

## Author contributions

DC, SL, ZF, and MM designed the study, DC and CE extracted the data, DC and MM performed the analyses. DC, MM, and ZF wrote the manuscript.

### Conflict of interest statement

The authors declare salary support for their research activity. The sponsors had no role in the design, analysis or reporting of the results. The views expressed by the authors do not reflect any official position of the government of the United States. The authors have no other relevant conflicts to report.

## References

[1] Fleischhacker WW Siu CO Boden R Pappadopulos E Karayal ON Kahn RS. Metabolic risk factors in first-episode schizophrenia: baseline prevalence and course analysed from the European First-Episode Schizophrenia Trial. Int J Neuropsychopharmacol. (2013) 16:987–95. 10.1017/S1461145712001241 23253821

[2] Laursen TM Munk-Olsen T Nordentoft M Mortensen PB. Increased mortality among patients admitted with major psychiatric disorders: a register-based study comparing mortality in unipolar depressive disorder, bipolar affective disorder, schizoaffective disorder, and schizophrenia. J. Clin. Psychiatry (2007) 68:899–907. 17592915 10.4088/jcp.v68n0612

[3] Weiner M Warren L Fiedorowicz JG. Cardiovascular morbidity and mortality in bipolar disorder. Ann Clin Psychiatry (2011) 23:40–7. 21318195 PMC3190964

[4] Goldstein BI Schaffer A Wang S Blanco C. Excessive and premature new-onset cardiovascular disease among adults with bipolar disorder in the US NESARC cohort. J Clin Psychiatry (2015) 76:163–9. 10.4088/JCP.14m09300 25742203

[5] Perry BI McIntosh G Weich S Singh S Rees K. The association between first-episode psychosis and abnormal glycaemic control: systematic review and meta-analysis. Lancet Psychiatry (2016) 3:1049–58. 10.1016/S2215-0366(16)30262-0 27720402

[6] Pillinger T Beck K Gobjila C Donocik JG Jauhar S Howes OD. Impaired glucose homeostasis in first-episode schizophrenia: a systematic review and meta-analysis. JAMA Psychiatry (2017) 74:261–9. 10.1001/jamapsychiatry.2016.3803 28097367 PMC6352957

[7] Burghardt KJ Seyoum B Mallisho A Burghardt PR Kowluru RA Yi Z. Atypical antipsychotics, insulin resistance and weight; a meta-analysis of healthy volunteer studies. Prog Neuro-psychopharmacology Biol Psychiatry (2018) 83:55–63. 10.1016/j.pnpbp.2018.01.004 29325867 PMC5817633

[8] Teff KL Rickels MR Grudziak J Fuller C Nguyen HL Rickels K. Antipsychotic-induced insulin resistance and postprandial hormonal dysregulation independent of weight gain or psychiatric disease. Diabetes (2013) 62:3232–40. 10.2337/db13-0430 23835329 PMC3749337

[9] Lieberman JA Stroup TS McEvoy JP Swartz MS Rosenheck RA Perkins DO. Effectiveness of antipsychotic drugs in patients with chronic schizophrenia. New Engl J Med. (2005) 353:1209–23. 10.1056/NEJMoa051688 16172203

[10] Lamia KA Storch KF Weitz CJ. Physiological significance of a peripheral tissue circadian clock. Proc Natl Acad Sci USA. (2008) 105:15172–7. 10.1073/pnas.0806717105 18779586 PMC2532700

[11] Marcheva B Ramsey KM Buhr ED Kobayashi Y Su H Ko CH. Disruption of the clock components CLOCK and BMAL1 leads to hypoinsulinaemia and diabetes. Nature (2010) 466:627–31. 10.1038/nature09253 20562852 PMC2920067

[12] Kettner NM Mayo SA Hua J Lee C Moore DD Fu L. Circadian dysfunction induces leptin resistance in mice. Cell Metab. (2015) 22:448–59. 10.1016/j.cmet.2015.06.005 26166747 PMC4558341

[13] Perelis M Marcheva B Ramsey KM Schipma MJ Hutchison AL Taguchi A. Pancreatic beta cell enhancers regulate rhythmic transcription of genes controlling insulin secretion. Science (2015) 350:aac4250. 10.1126/science.aac4250 26542580 PMC4669216

[14] Freyberg Z McCarthy MJ. Dopamine D2 receptors and the circadian clock reciprocally mediate antipsychotic drug-induced metabolic disturbances. NPJ Schizophrenia (2017) 3:17. 10.1038/s41537-017-0018-4 28560263 PMC5441531

[15] Panda S. Circadian physiology of metabolism. Science (2016) 354:1008–15. 10.1126/science.aah4967 27885007 PMC7261592

[16] Polonsky KS Given BD Hirsch LJ Tillil H Shapiro ET Beebe C. Abnormal patterns of insulin secretion in non-insulin-dependent diabetes mellitus. New Engl J Med. (1988) 318:1231–9. 10.1056/NEJM198805123181903 3283554

[17] Chung S Lee EJ Yun S Choe HK Park SB Son HJ. Impact of circadian nuclear receptor REV-ERBalpha on midbrain dopamine production and mood regulation. Cell (2014) 157:858–68. 10.1016/j.cell.2014.03.039 24813609

[18] Mendoza J Challet E. Circadian insights into dopamine mechanisms. Neuroscience (2014) 282:230–42. 10.1016/j.neuroscience.2014.07.081 25281877

[19] Ozburn AR Falcon E Twaddle A Nugent AL Gillman AG Spencer SM. Direct regulation of diurnal Drd3 expression and cocaine reward by NPAS2. Biol Psychiatry (2015) 77:425–33. 10.1016/j.biopsych.2014.07.030 25444159 PMC4315729

[20] Simpson N Maffei A Freeby M Burroughs S Freyberg Z Javitch J. Dopamine-mediated autocrine inhibitory circuit regulating human insulin secretion *in vitro*. Mol Endocrinol. (2012) 26:1757–72. 10.1210/me.2012-1101 22915827 PMC3458225

[21] Sugai T Suzuki Y Fukui N Watanabe J Ono S Tsuneyama N. Excessive insulin secretion in Japanese schizophrenic patients treated with antipsychotics despite normal fasting glucose levels. J Clin Psychopharmacol. (2012) 32:750–5. 10.1097/JCP.0b013e3182742ea4 23131894

[22] Ballon JS Pajvani U Freyberg Z Leibel RL Lieberman JA. Molecular pathophysiology of metabolic effects of antipsychotic medications. Trends Endocrinol Metab (2014) 25:593–600. 10.1016/j.tem.2014.07.004 25190097

[23] Perez-Iglesias R Ortiz-Garcia de la Foz V Martinez Garcia O Amado JA Garcia-Unzueta MT Ayesa-Arriola R. Comparison of metabolic effects of aripiprazole, quetiapine and ziprasidone after 12 weeks of treatment in first treated episode of psychosis. Schizophrenia Res. (2014) 159:90–4. 10.1016/j.schres.2014.07.045 25151200

[24] Nguyen C Novac A Hazen J Howard P Tieu R Bota RG. Weight gain changes in patients with aripiprazole monotherapy compared with aripiprazole-antidepressant polypharmacy in an outpatient sample. Journal of psychopharmacology (2017) 32:423–29. 10.1177/0269881117742659 29215304

[25] Vazquez-Bourgon J Perez-Iglesias R Ortiz-Garcia de la Foz V Suarez Pinilla P Diaz Martinez A Crespo-Facorro B. Long-term metabolic effects of aripiprazole, ziprasidone and quetiapine: a pragmatic clinical trial in drug-naive patients with a first-episode of non-affective psychosis. Psychopharmacology (2018) 235:245–55. 10.1007/s00213-017-4763-x 29075885

[26] Freyberg Z Aslanoglou D Shah R Ballon JS. Intrinsic and antipsychotic drug-induced metabolic dysfunction in schizophrenia. Front Neurosci. (2017) 11:432. 10.3389/fnins.2017.00432 28804444 PMC5532378

[27] Garcia-Tornadu I Ornstein AM Chamson-Reig A Wheeler MB Hill DJ Arany E. Disruption of the dopamine d2 receptor impairs insulin secretion and causes glucose intolerance. Endocrinology (2010) 151:1441–50. 10.1210/en.2009-0996 20147524

[28] Borcherding DC Hugo ER Idelman G De Silva A Richtand NW Loftus J. Dopamine receptors in human adipocytes: expression and functions. PLoS ONE (2011) 6:e25537. 10.1371/journal.pone.0025537 21966540 PMC3180449

[29] Kok P Roelfsema F Frolich M van Pelt J Meinders AE Pijl H. Activation of dopamine D2 receptors lowers circadian leptin concentrations in obese women. J. Clin. Endocrinol. Metab. (2006) 91:3236–40. 10.1210/jc.2005-2529 16705078

[30] Kok P Roelfsema F Frolich M van Pelt J Stokkel MP Meinders AE. Activation of dopamine D2 receptors simultaneously ameliorates various metabolic features of obese women. Am J Physiol Endocrinol Metab. (2006) 291:E1038–43. 10.1152/ajpendo.00567.2005 16803851

[31] Duffy D Rader DJ. Update on strategies to increase HDL quantity and function. Nature Rev Cardiol. (2009) 6:455–63. 10.1038/nrcardio.2009.94 19488077

[32] Rashid S Watanabe T Sakaue T Lewis GF. Mechanisms of HDL lowering in insulin resistant, hypertriglyceridemic states: the combined effect of HDL triglyceride enrichment and elevated hepatic lipase activity. Clin Biochem. (2003) 36:421–9. 12951168 10.1016/s0009-9120(03)00078-x

[33] van den Berg R Noordam R Kooijman S Jansen SWM Akintola AA Slagboom PE. Familial longevity is characterized by high circadian rhythmicity of serum cholesterol in healthy elderly individuals. Aging Cell (2017) 16:237–43. 10.1111/acel.12547 28440906 PMC5334529

[34] Osonoi Y Mita T Osonoi T Saito M Tamasawa A Nakayama S. Morningness-eveningness questionnaire score and metabolic parameters in patients with type 2 diabetes mellitus. Chronobiol Int. (2014) 31:1017–23. 10.3109/07420528.2014.943843 25102425

[35] Knutson KL von Schantz M. Associations between chronotype, morbidity and mortality in the UK Biobank cohort. Chronobiol Int. (2018) 35:1045–53. 10.1080/07420528.2018.1454458 29642757 PMC6119081

[36] Di Sciascio G Riva MA. Aripiprazole: from pharmacological profile to clinical use. Neuropsychiatric Dis Treatment (2015) 11:2635–47. 10.2147/NDT.S88117 26508859 PMC4610784

[37] Takiguchi T Tomita M Matsunaga N Nakagawa H Koyanagi S Ohdo S. Molecular basis for rhythmic expression of CYP3A4 in serum-shocked HepG2 cells. Pharmacogenet Genomics (2007) 17:1047–56. 10.1097/FPC.0b013e3282f12a61 18004209

[38] Matsunaga N Inoue M Kusunose N Kakimoto K Hamamura K Hanada Y. Time-dependent interaction between differentiated embryo chondrocyte-2 and CCAAT/enhancer-binding protein alpha underlies the circadian expression of CYP2D6 in serum-shocked HepG2 cells. Mol Pharmacol. (2012) 81:739–47. 10.1124/mol.111.076406 22355045

[39] Shapiro DA Renock S Arrington E Chiodo LA Liu LX Sibley DR. Aripiprazole, a novel atypical antipsychotic drug with a unique and robust pharmacology. Neuropsychopharmacology (2003) 28:1400–11. 10.1038/sj.npp.1300203 12784105

